# Correction: Ocular biocompatibility evaluation of POSS nanomaterials for biomedical material applications

**DOI:** 10.1039/d4ra90030h

**Published:** 2024-03-28

**Authors:** Chenghui Shen, Yuemei Han, Bailiang Wang, Junmei Tang, Hao Chen, Quankui Lin

**Affiliations:** a School of Ophthalmology & Optometry, Eye Hospital, Wenzhou Medical University Wenzhou 325027 China Chenhao823@mail.eye.ac.cn lqk97531@126.com +86-577-8806-7962; b Wenzhou Institute of Biomaterials and Engineering Wenzhou 325000 China

## Abstract

Correction for ‘Ocular biocompatibility evaluation of POSS nanomaterials for biomedical material applications’ by Chenghui Shen *et al.*, *RSC Adv.*, 2015, **5**, 53782–53788, https://doi.org/10.1039/C5RA08668J.

The authors regret that the magnification of the cell staining images was missed in the caption of Fig. 4. The corrected caption of Fig. 4 is as shown here. The figures remain the same.

**Fig. 4** Fluorescence micrographs of DCFH-DA stained HLECs (A), and cells incubated with 200 mg mL^−1^ NH_2_-POSS (B), HO-POSS (C) or SH-POSS (D), for indicating the intracellular ROS level (200×).

The Royal Society of Chemistry apologises for these errors and any consequent inconvenience to authors and readers.

## Supplementary Material

